# Cross sectional analysis of respiratory symptoms in an injection drug user cohort: the impact of obstructive lung disease and HIV

**DOI:** 10.1186/1471-2466-10-27

**Published:** 2010-05-11

**Authors:** M Bradley Drummond, Gregory D Kirk, Erin P Ricketts, Meredith C McCormack, J Christian Hague, John F McDyer, Shruti H Mehta, Eric A Engels, Robert A Wise, Christian A Merlo

**Affiliations:** 1 Department of Medicine, School of Medicine, Johns Hopkins University, Baltimore, MD, USA; 2 Department of Epidemiology, Bloomberg School of Public Health, Johns Hopkins University, Baltimore, MD, USA; 3 Division of Cancer Epidemiology and Genetics, National Cancer Institute, National Institutes of Health, Rockville, MD, USA

## Abstract

**Background:**

Injection drug use is associated with an increased risk of human immunodeficiency virus (HIV) infection and with obstructive lung diseases (OLD). Understanding how HIV and OLD may impact respiratory symptoms among injection drug users (IDUs) is important to adequately care for this high-risk population. We characterized the independent and joint effects of HIV and OLD on respiratory symptoms of a cohort of inner-city IDUs.

**Methods:**

Demographics, risk behavior and spirometric measurements were collected from a cross-sectional analysis of the Acquired Immunodeficiency Syndrome Link to the IntraVenous Experience study, an observational cohort of IDUs followed in Baltimore, MD since 1988. Participants completed a modified American Thoracic Society respiratory questionnaire and the Medical Research Council (MRC) dyspnea score to assess respiratory symptoms of cough, phlegm, wheezing and dyspnea.

**Results:**

Of 974 participants, 835 (86%) were current smokers and 288 (29.6%) were HIV-infected. The prevalence of OLD (FEV1/FVC ≤ 0.70) was 15.5%, and did not differ by HIV status. OLD, but not HIV, was associated with increased frequency of reported respiratory symptoms. There was a combined effect of OLD and HIV on worsening of MRC scores. OLD and HIV were independently associated with an increased odds of reporting an MRC ≥ 2 (OR 1.83 [95%CI 1.23-2.73] and 1.50 [95%CI 1.08-2.09], respectively). COPD, but not HIV, was independently associated with reporting an MRC ≥ 3 (OR 2.25 [95%CI 1.43-3.54] and 1.29 [95%CI 0.87-1.91], respectively).

**Conclusions:**

While HIV does not worsen cough, phlegm or wheezing, HIV significantly increases moderate but not severe dyspnea in individuals of similar OLD status. Incorporating the MRC score into routine evaluation of IDUs at risk for OLD and HIV provides better assessment than cough, phlegm and wheezing alone.

## Background

Injection drug use (IDU) is a prevalent social behavior in urban centers that is associated with many acute and chronic medical illnesses [1-4]. Due to similar underlying risk factors in injection drug users (IDUs), unique chronic medical conditions frequently co-exist in the same individual [5-10]. IDU is a risk factor for the development of two prevalent diseases: obstructive lung diseases (OLD), specifically chronic obstructive pulmonary disease (COPD) [11-13]. and human immunodeficiency virus (HIV) infection[3,14,15]. In 2006, nearly 140,000 deaths were attributed to either COPD or HIV in the States[16,17].

Risk factors for the development of OLD, such as tobacco use and low socio-economic status, overlap in those with HIV[18]. Prior studies have described the frequent coexistence of HIV and OLD in at-risk populations[11,19,20]. Because IDUs are particularly vulnerable to both OLD and HIV, it is important to explore how these two diseases may impact respiratory status. To date, no data exist on the impact of OLD and HIV on respiratory symptoms and functional status among and IDU population. The Acquired Immunodeficiency Syndrome (AIDS) Link to IntraVenous Experience (ALIVE) study[21]. has prospectively observed a cohort of both HIV-infected and at risk IDUs in inner-city Baltimore, Maryland, since 1988, providing an ideal dataset to examine the independent and joint effects of HIV and OLD on manifestation of respiratory disease.

## Methods

### Setting and Participants

The methods for recruitment and data collection in the ALIVE study have been previously described[21]. Subjects were recruited through community outreach programs outside of treatment using distribution of flyers and word of mouth. Briefly, participants were required to be ≥ 18 yrs of age and have a history of injecting drugs. ALIVE participants that underwent spirometry testing as part of the lung sub-study were included in the current analysis. This study was approved by Institutional Review Boards of the Johns Hopkins Bloomberg School of Public Health and the National Cancer Institute. All enrolled participants provided written informed consent.

### Data Collection

In this cross-sectional analysis, exposure and outcome data were obtained from the ALIVE study visit where spirometry was first obtained; participants also underwent clinical examination, collection of blood specimens, behavior and respiratory questionnaires. Smoking status and duration, recent injection drug use and anti-retroviral or bronchodilator therapy were determined by self-report. Medical conditions such as respiratory infections or clinical AIDS events were identified through self-report and confirmed through standardized medical record abstraction. Spirometry was performed using a KOKO^® ^(Pulmonary Data Services, Inc., Louisville, CO) pneumotach in accordance with American Thoracic Society guidelines[22]. Percent predicted values were calculated using standard formulas[23]. OLD was defined as a pre-bronchodilator ratio of the forced expiratory volume in one second (FEV1) to forced vital capacity (FVC) of ≤ 70%[24]. Participants completed a modified version of the American Thoracic Society respiratory questionnaire[25]. Our questionnaire captured information regarding presence, frequency and timing of respiratory symptoms (cough, phlegm and wheezing). Dyspnea was assessed using the modified Medical Research Council (MRC) questionnaire with a validated 0-4 scale, with a higher score indicating worse dyspnea[26,27]. The MRC score has been shown to correlate with lung function and long-term mortality in COPD patients and thus is employed as a tool for assessment of disease impact [27-29].

### Statistical analysis

We compared the prevalence of respiratory symptoms in individuals with and without OLD (defined by FEV1/FVC ≤ 70%) grouped by HIV status. Clinical and demographic characteristics between groups were compared using t-test for continuous variables. Categorical variables were compared with Pearson's chi square and Fisher's exact tests. Analysis of variance was performed to test the effects of HIV and OLD on clinical and spirometric measurements. Because of multiple comparisons, a Bonferroni corrected p-value was used to assess for statistical significance in the two-way comparison. Differences in the distribution of MRC scores were assessed with Pearson's chi squared test. Multivariable logistic regression models were generated to explore combinations of variables associated with different MRC thresholds. Covariates were evaluated based upon known relevance from clinical literature review and/or inspection of exploratory data analysis. Odds ratios were adjusted for age, gender, body-mass index (BMI), lifetime pack-years smoking, number of lifetime pneumonias, presence of severe anemia (hemoglobin<8 mg/dL) and injection drug use status. Hemoglobin samples were not collected on HIV-uninfected participants prior to October 2007, thus these values were missing on 469 (48.2%) of the cohort. Results with and without this covariate in logistic regression modeling did not affect the overall results. Additional multivariable models restricted to HIV-infected individuals compared thresholds of viral load and CD4 levels. All data are presented as mean (standard deviation) for normally distributed data and median (interquartile range [IQR]) for non-normally distributed data. Stata version 10.0 (Stata Corp, College Station, TX), was used for statistical analysis.

## Results

### Baseline Characteristics

Of the 1052 participants evaluated in the lung sub-study, 78 participants lacked complete spirometric data and were excluded. Therefore, analysis presented includes demographic, anthropometric, spirometric and respiratory questionnaire data from 974 individuals. Overall, participants were a median age of 48.7 years old (IQR, 43.6-53.5 years), 65.9% were males, 89.8% were black. Overall, 85.7% were current smokers, 9.3% were former smokers and 4.9% had never smoked. A total of 288 (29.6%) individuals were HIV-infected. Of HIV-infected participants, 155 (54.6%) reported highly active anti-retroviral therapy (HAART) use in the last 6 months. HIV-infected HAART users had a median CD4 count was 322 cells/mm^3 ^(IQR, 177-503) and 109 (70.3%) had an undetectable viral load. Of the 133 HIV-infected individuals not reporting HAART use in the last 6 months, the median CD4 count was 321 cells/mm^3 ^(IQR, 178-497) and 22 (16.5%) had an undetectable viral load. The prevalence of HIV infection among the 78 excluded participants (30.8%) was similar to those included in analysis (29.6%).

The prevalence of spirometry-defined OLD among participants was 15.5%, which did not differ by HIV status (HIV+ 15.6% vs. HIV-15.5%; p = 0.95). Study groups formed by the presence or absence of OLD and HIV included 580 participants negative for both diseases, 243 participants with HIV alone, 106 participants with OLD alone and 45 participants with both OLD and HIV (Table 1). OLD+/HIV-participants were slightly older than the other disease subgroups. Distribution of race differed among the four disease strata. OLD-/HIV+ individuals were more likely to be black when compared to those with neither disease (OLD-/HIV-) (96.3 vs. 88.3%; p < 0.01) and both diseases (OLD+/HIV+) (96.3 vs. 86.7%; p = 0.048). BMI also differed with HIV and COPD status. Among HIV-participants, the presence of OLD was associated with a lower BMI when compared to those without COPD (p = 0.04). The presence of HIV was not associated with a difference in BMI among individuals of similar OLD status. As expected, bronchodilator use was more common among patients with OLD regardless of HIV status (OLD+/HIV-vs. OLD-/HIV-33.0 vs. 12.1%; p < 0.01 and OLD+/HIV+ vs. OLD-/HIV+ 44.4 vs. 19.3%; p < 0.01). In participants without OLD, bronchodilator use was more frequently reported when HIV was present compared to when HIV was absent (19.3% vs. 12.1%; p < 0.01).

**Table 1:** Clinical and demographic characteristics of study participants.

	OLD-		OLD+		
	**HIV-**	**HIV+**	**HIV-**	**HIV+**	P-value*

N	580	243	106	45	

Age, yr	48.1 (8.04)	47.7 (6.44)	50.1 (8.82)	48.8 (8.40)	0.05

Male, n(%)	385 (66.4)	152 (62.6)	75 (70.8)	30 (66.7)	0.49

Race/ethnicity, n(%)					
Black	512 (88.3)	234 (96.3)	90 (84.9)	39 (86.7)	<0.01

Smoking status, n(%)					
Current	500 (86.2)	203 (83.5)	94 (88.7)	38 (84.4)	
Former	53 (9.14)	25 (10.3)	10 (9.43)	3 (6.67)	
Never	27 (4.66)	15 (6.17)	2 (1.89)	4 (8.89)	0.53

Smoking, pack-yr	19.5 (12-33)	19 (10.5-32.4)	20.8 (14-36)	21.9 (10.8-36.5)	0.56

Current IDU, n(%)	246 (42.4)	87 (35.8)	44 (41.5)	15 (33.3)	0.25

6 mo income, n(%)					
Not reported	12 (2.1)	7 (2.9)	5 (4.7)	2 (4.4)	
None	147 (25.3)	52 (21.4)	21(19.8)	7 (15.6)	
<$5,000	274 (47.2)	133 (54.7)	51 (48.1)	25 (55.6)	
$5,000-$10,000	91 (15.7)	39 (16.0)	23 (21.7)	6 (13.3)	
>$10,000	56 (9.7)	12 (4.9)	6 (5.7)	5 (11.1)	0.13

High school education, n(%)	259 (44.7)	85 (35.0)	42 (39.6)	18 (45)	0.10

BMI, (kg/m^2^)	25.7 (22.8-30.3)	24.9 (21.8-29.1)	24.2 (22.0-27.6)	22.3 (21.2-28.3)	<0.01

Hemoglobin (gm/dL)	13.4 (1.61)	12.8 (1.58)	13.4 (1.82)	12.9 (1.91)	<0.01

MRC score	0.96 (1.25)	1.19 (1.31)	1.35 (1.41)	1.69 (1.46)	<0.01

Bronchodilator use, n(%)	70 (12.1)	47 (19.3)	35 (33.0)	20 (44.4)	<0.01

CD_4 _cell count	N/A	323 (177-502)	N/A	305 (168-487)	0.88

HIV viral load					
>400 copies, n(%)	N/A	112 (46.1)	N/A	19 (42.2)	0.63
X 100K, mean(SD)^±^		62.2 (103)		111 (176)	0.06

HAART use, n(%)	N/A	133 (54.7)	N/A	22 (48.9)	0.19

Pneumonia, n(%)					
All	15 (2.59)	3 (1.23)	3 (2.83)	1 (2.22)	0.57
*Pneumocystis*	0	2 (0.82)	0	0	0.22

*P-value represents test for overall difference across means or proportions. Values presented as mean (S.D.) or median (IQR) unless indicated. OLD = Obstructive lung disease; HIV = Human immunodeficiency virus; IDU = Injection drug use; BMI = Body mass index; MRC = Medical research council; CD4 = CD4+ T-Helper cells; HAART = Highly active anti-retroviral therapy; pack-yr = Number of packs smoked per day * years smoked; ^±^Mean(SD) of viral load only for those participants with viral load >400.

### Lung function

Among participants with OLD, those with HIV infection had a lower absolute FEV1 and FVC compared to those without HIV (1.97 L [0.73] vs. 2.36 L [0.84]; p = 0.019 and 3.17 L [1.01] vs. 3.69 L [1.17]; p = 0.014, respectively) (Table 2). However, in those individuals with OLD, there was no difference by HIV status in percent predicted FEV1 (FEV1% predicted) (p = 0.05) and percent predicted FVC (FVC% predicted) (p = 0.15).

**Table 2:** Spirometry characteristics of study participants.

	OLD-		OLD+		
	**HIV-**	**HIV+**	**HIV-**	**HIV+**	P-value*

FEV_1_/FVC	79.2 (4.83)	79.8 (5.44)	63.4 (6.75)	61.2 (7.95)	<0.01

FEV_1_					
Absolute (L)	2.93 (0.75)	2.80 (0.66)	2.36 (0.84)	1.97 (0.73)	<0.01
% Predicted	95.9 (15.3)	95.0 (15.2)	73.8 (18.2)	66.4 (19.7)	<0.01

FVC					
Absolute (L)	3.71 (0.96)	3.53 (0.86)	3.69 (1.17)	3.17 (1.01)	<0.01
% Predicted	97.5 (15.8)	96.2 (15.8)	92.3 (18.9)	86.1 (20.5)	<0.01

*P-value represents test for overall difference across means. Values presented as mean (S.D.) OLD = Obstructive lung disease; HIV = human immunodeficiency virus; FEV1 = Forced expiratory volume in 1 sec; FVC = Forced vital capacity;

### Respiratory Symptoms

In individuals with neither OLD nor HIV, respiratory symptoms were commonly reported, with 25.5% reporting cough, 29.5% reporting phlegm and 34.5% reporting wheezing (Table 3). OLD, but not HIV, was associated with more frequent respiratory symptoms. For HIV-participants, those with OLD reported more frequent symptoms than those without OLD: cough 50.9% vs. 25.5% (p < 0.01), phlegm 49.1% vs. 29.5% (p < 0.01) and wheezing 61.3% vs. 34.5% (p < 0.01). Similarly, for HIV+ participants, those with OLD reported more frequent respiratory symptoms when compared to those without OLD: cough 53.3% vs. 26.3% (p < 0.01), phlegm 60.0 vs. 31.3% (p < 0.01) and wheezing 60.0 vs. 35.8% (p < 0.01). When evaluating only HIV-infected subjects, neither HAART use nor undetectable HIV RNA levels were associated with differences in respiratory symptoms in individuals of similar OLD status.

**Table 3:** Prevalence of respiratory symptoms stratified by OLD and HIV status.

	OLD-		OLD+	
	**HIV-**	**HIV+**	**HIV-**	**HIV+**

N	580	243	106	45

Cough, n(%)				
Present	148 (25.5)	64 (26.3)	54 (50.9)	24 (53.3)
Morning cough	93 (16.0)	38 (15.6)	35 (33.0)	17 (37.8)
≥ 4 days/week	109 (18.8)	46 (18.9)	37 (34.9)	23 (51.1)
≥ 3 months	61 (10.5)	31 (12.8)	22 (20.8)	11 (24.4)

Phlegm, n(%)				
Present	171 (29.5)	76 (31.3)	52 (49.1)	27 (60.0)
Morning phlegm	101 (17.4)	44 (18.1)	39 (36.8)	18 (40.0)
≥ 4 days/week	109 (18.8)	54 (22.2)	37 (34.9)	21 (46.7)
≥ 3 months	78 (13.5)	30 (12.4)	24 (22.6)	13 (28.9)

Wheezing, n(%)				
Ever	200 (34.5)	87 (35.8)	65 (61.3)	27 (60.0)
Most days and nights	61 (10.5)	31 (12.8)	30 (28.3)	15 (33.3)
History of wheezing attack	117 (20.2)	54 (22.2)	47 (44.3)	21 (46.7)

Values presented as n(%). OLD = Obstructive lung disease; HIV = human immunodeficiency virus

**Figure 1:**
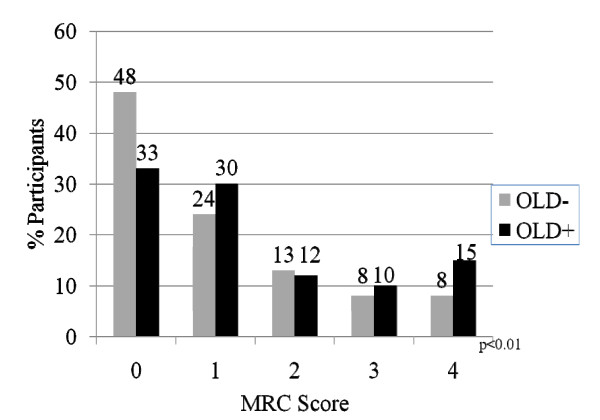
**Distribution of MRC scores by disease status**. The distribution of MRC scores by OLD status alone (OLD = Obstructive lung disease; MRC = Medical research council). P-values represent overall chi-squared test.

**Figure 2:**
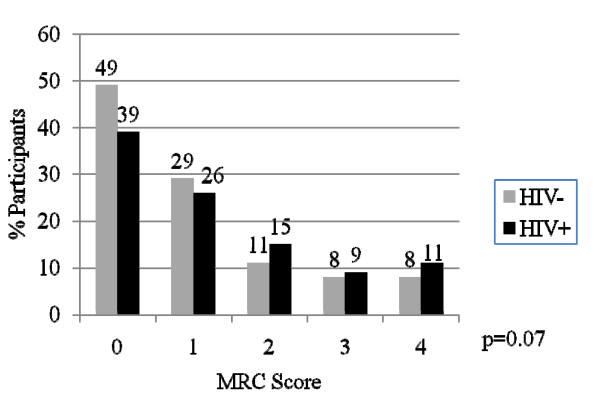
**Distribution of MRC scores by disease status**. The distribution of MRC scores by HIV status alone. (HIV = Human immunodeficiency virus; MRC = Medical research council). P-values represent overall chi-squared test.

**Figure 3:**
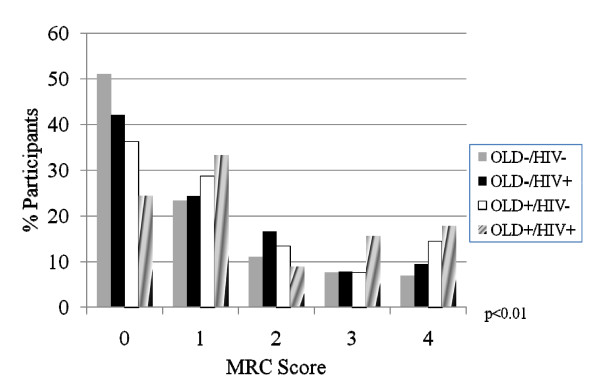
**Distribution of MRC scores by disease status**. The distribution of MRC scores by combined OLD and HIV states. (OLD = Obstructive lung disease; HIV = Human immunodeficiency virus; MRC = Medical research council). P-values represent overall chi-squared test.

In order to assess the independent and joint effects of HIV and OLD on dyspnea, we examined the distribution of MRC scores by disease subgroup. Additionally, we used logistic regression analysis to determine characteristics associated with MRC score thresholds of ≥ 2 and ≥ 3. These thresholds were chosen because of their known correlation with significant impairment in functional status (≥ 2)[30]. and with mortality (≥ 3)[28,31]. When stratifying by OLD alone, the distribution of MRC scores was worse for OLD+ individuals when compared to OLD-individuals (p < 0.01) (Figure 1). When stratifying by HIV status alone, the distribution of MRC scores trended towards worse scores for HIV+ individuals when compared to HIV-individuals, although the test for trend did not achieve statistical significance (p = 0.07) (Figure 2). The distribution of MRC scores by each of the OLD/HIV groups approximated an additive effect of HIV and OLD (p < 0.01) (Figure 3). An MRC ≥ 2 was present in 151 (25.9%) of OLD-/HIV-individuals, 82 (33.1%) of OLD-/HIV+ participants, 38 (35.8%) of OLD+/HIV-individuals and 19 (42.2%) of OLD+/HIV+ participants (overall chi-square p = 0.01). An MRC ≥ 3 was present in 87 (14.9%) of OLD-/HIV-participants, 42 (16.9%) of OLD-/HIV+ participants, 24 (22.6%) of OLD+/HIV-participants and 15 (33.3%) of OLD+/HIV+ participants (overall p < 0.01). Among HIV-infected individuals, neither CD4 cell count<200, HAART use, nor undetectable HIV RNA levels were associated with a difference in the distribution and mean MRC scores in individuals of similar OLD status.

After adjusting for demographic characteristics, smoking intensity, current injection drug use and number of pneumonias, logistic regression analysis demonstrated that OLD was associated with an increased odds of reporting an MRC ≥ 2 (OR 1.83; 95%CI 1.23-2.73; p < 0.01) and an MRC ≥ 3 (OR 2.25; 95%CI 1.43-3.54; p < 0.01) (Table 4). HIV infection was associated with an increased odds of reporting an MRC ≥ 2 (OR 1.50; 95%CI 1.08-2.09; p = 0.02) but this association was attenuated for an MRC ≥ 3 (OR 1.29; 95%CI 0.87-1.91; p = 0.20). Female gender, higher BMI and higher lifetime pack-years smoking were associated with an increase in the odds of reporting an MRC ≥ 2 and ≥ 3 in all models. In HIV-infected subjects, adjusting for HIV severity (viral load>200,000 copies/mL versus viral load ≤ 200,000 copies/mL or CD4>200 vs. CD4 ≤ 200), did not attenuate the effect of OLD on dyspnea.

**Table 4:** Adjusted odds ratio (95%CI) of MRC thresholds*.

	MRC ≥ 2	MRC ≥ 3
OLD	1.83 (1.23-2.73)	2.25 (1.43-3.54)

HIV	1.50 (1.08-2.09)	1.29 (0.87-1.91)

Female	3.86 (2.81-5.30)	4.38 (2.99-6.42)

BMI (per kg/m^2^)	1.06 (1.03-1.08)	1.05 (1.02-1.07)

Smoking (per 10-pk yrs)	1.24 (1.13-1.36)	1.22 (1.09-1.36)

*Adjusted for other variables in the table as well as age, current injection drug use status and number of pneumonias. 95%CI = 95% confidence interval; OLD = Obstructive lung disease; HIV = Human immunodeficiency virus; BMI = Body mass index; pack-yr = Number of packs smoked per day * years smoked

## Discussion

In this report, we have characterized the respiratory symptoms of a large cohort of IDUs at-risk for HIV and OLD. This analysis has yielded several important findings. First, in this population with almost ubiquitous cigarette smoking, we identified a high prevalence of respiratory symptoms even in the absence of OLD and HIV. While there was not an increased prevalence of reported cough, phlegm and wheezing associated with HIV, we did find that when HIV infection and OLD coexisted, dyspnea was worse than when either disease was present alone. Furthermore, HIV was independently associated with moderate dyspnea after controlling for the presence of OLD and other confounders.

Within this IDU cohort, a high prevalence of respiratory symptoms was present even in those participants without OLD or HIV. Despite the mean age of 48.7 years in our study cohort, the rates of respiratory symptoms observed in those without OLD and HIV are similar to rates reported in a population of elderly Medicare smokers[32]. A prior report of 234 HIV-infected individuals, of whom 6.8% had spirometry-defined COPD, described a similar frequency of respiratory symptoms[33]. Our report highlights the significant symptom burden experienced by the IDU population with heavy tobacco habits. We extend prior publications by stratifying respiratory symptoms and functional status in a population of injection drug users by OLD and HIV status. Prior publications have been limited to descriptions of respiratory symptoms in either OLD or HIV, but not in both disease states[34,35].

While HIV status was not associated with increasing cough, phlegm and wheezing, there was a combined effect of HIV and OLD on dyspnea assessed by the MRC scale. To our knowledge, no prior study has evaluated the effect of HIV on MRC scores in individuals with and without OLD. Our analysis has shown that the presence of HIV is associated with a detrimental shift in the distribution of MRC scores when OLD is present, and this effect was independent of HIV severity. Moreover, patients who had both OLD and HIV had the highest proportion of MRC scores ≥ 2 and ≥ 3. Female gender was associated with increased odds of reporting an MRC ≥ 2 and ≥ 3 after adjusting for other characteristics. These findings add information to prior publications examining respiratory symptoms in HIV-infected populations,[11] which have not included female participants. We observed a >50% increase in the risk of reporting an MRC ≥ 2 in the presence of HIV, highlighting the impact of HIV infection on dyspnea of individuals without substantially altering cough, phlegm and wheezing. Interestingly, OLD, but not HIV, remained an important predictor of reporting an MRC ≥ 3, suggesting that HIV may increase mild impairment independent of OLD, but advanced dyspnea is largely driven by OLD.

Several potential mechanisms exist by which HIV infection may increase the susceptibility to respiratory symptoms and diseases. HIV-infected individuals demonstrate elevated systemic inflammatory cytokines, including those implicated in the pathogenesis of OLD [36-38]. Moreover, elevated inflammatory cytokines are observed in the bronchoalveolar lavage fluid of asymptomatic HIV-infected individuals[39]. In lung autopsy specimens of HIV-infected individuals, areas of histological emphysema had many HIV-1 infected cells, whereas rare HIV-1 infected cells were evident in normal lung, suggesting that direct HIV infection may drive emphysema formation[40]. These observations suggest that infection with HIV leads to systemic and local abnormalities that may promote the development of OLD. Additionally, HIV infection is associated with an increase in the development of pulmonary arterial hypertension,[41] which may be independently associated with increased respiratory symptoms.

Our study has several implications for health care providers caring for IDU patients at risk for OLD and HIV. The high prevalence of smoking and respiratory symptoms identifies IDUs as patients who would benefit from aggressive smoking cessation and spirometry screening initiatives. Second, when assessing respiratory symptoms in HIV+ patients at-risk for OLD, providers should be hesitant to attribute reported respiratory symptoms solely to HIV infection. Rather, healthcare providers should be vigilant to screen for undiagnosed OLD in this population. Third, clinicians should consider incorporating dyspnea as well as cough, phlegm and wheezing into routine assessments. Given that MRC scores have been shown to correlate with morbidity and mortality in longitudinal studies of OLD patients,[28,31] incorporation of the MRC scale into routine clinical assessments of IDUs may provide further risk stratification of populations with the combined co-morbidities of OLD and HIV.

Our study has some limitations. First, the cross-sectional nature of this analysis prevents conclusions regarding longitudinal outcomes such as rate of FEV1 decline and mortality. Although analyses incorporating markers of advance HIV did not attenuate the effect of OLD on respiratory symptoms, the high background prevalence of smoking and respiratory symptoms in our population may be obscuring any effect of advanced HIV on respiratory illness. IDUs participating in longitudinal observational studies may differ from the general IDU population, impacting generalizability. Moreover, the inner-city characteristics of this cohort may further limit the generalizability of our findings to other IDU population or different populations with OLD and HIV. The absence of pre- and post-bronchodilator testing in this cohort does not allow us to make conclusions regarding the different symptom profile of asthma and COPD. While we accounted for number of pneumonias in our analysis, prior studies have reported that IDUs have an increased risk of bacterial pneumonia and tuberculosis when compared to other HIV risk groups, a confounder that could impact functional status[42,43]. We also did not evaluate for underlying cardiac disease as a contributor to dyspnea. Despite these limitations, the large size of our cohort, the systematic administration of validated questionnaires and the use of standardized measures of lung function allow us to provide a novel and comprehensive study of respiratory symptoms and functional status in an inner-city IDU population at risk for HIV and OLD.

## Conclusions

We show that HIV significantly worsens dyspnea, but has limited effect on the reported burden of cough, wheezing and phlegm in individuals of similar OLD status. Moreover, we found that OLD and HIV have an additive detrimental effect on dyspnea. HIV is independently associated with moderate but not severe dyspnea after controlling for OLD status. The results of this analysis provide new information which can assist providers managing a population particularly susceptible to the two important diseases of OLD and HIV.

## Abbreviations

AIDS: Acquired Immunodeficiency Syndrome; ALIVE: AIDS Linked to IntraVenous Experience; BMI: Body-mass index; CD4: CD4+ T-helper cells; COPD: Chronic obstructive pulmonary disease; FEV1: Forced expiratory volume in one second; FVC: Forced vital capacity; HAART: Highly active anti-retroviral therapy; HIV: Human immunodeficiency virus; IQR: Interquartile range; IDU: Injection drug use; IDUs: Injection drug users; MRC: Medical research council; OLD: Obstructive lung disease

## Competing interests

The authors declare that they have no competing interests.

## Authors' contributions

MBD is responsible for initial manuscript drafting, statistical analysis and revisions of the manuscript. GDK is responsible for initial research design and critical revisions of the manuscript. EPR is responsible for statistical analysis and critical revisions of the manuscript. MCM, JFM, SHM, EAE, RAW, CAM and JCH are responsible for the initial manuscript design and critical revisions of the manuscript. All authors read and approved the final manuscript.

## Pre-publication history

The pre-publication history for this paper can be accessed here:

http://www.biomedcentral.com/1471-2466/10/27/prepub
